# Supplementary material for: Molecular Biomarkers in Perthes Disease: A Review
Source: Diagnostics (Basel). 2023 Jan 27;13(3):471. doi: 10.3390/diagnostics13030471 (PMC9914190; doi:10.3390/diagnostics13030471)
Supplement: Supplementary file 1 [file diagnostics-13-00471-s001.zip › diagnostics-2136654-supplementary.pdf]

Protein sequence alignment of COL2A1 protein. COL2A1 protein show high conservation of primary structure among distant

Supplementary material

https://www.uniprot.org

```
CLUSTAL O(1.2.4) multiple sequence alignment

SP|P02458|CO2A1_HUMAN  MIRLGAPQTLVLL---TLLVAAVLRCQG-QDVQEAGSCVQDGQRYNDKDVWKPEPCRICV 56
SP|P28481|CO2A1_MOUSE  MIRLGAPQSLVLL---TLLIAAVLRCQG-QDAQEAGSCLQNGQRYKDKDVWKPSSCRICV 56
SP|Q91717|CO2A1_XENLA  MFSFVDSRTLVLFAATQVILLAVVRCQDEEDVLDTGSCVQHGQRYSDKDVWKPEPCQICV 60
TR|Q2LDA1|Q2LDA1_DANRE MFRLLDSRTLLLLVATHSVLLSLVRCQQEDDQEEFGGCVQDGQQYADRAVWKPEPCRVCV 60
                       *: :   ::*:*:     :: :::***  :*  : *.*:*.**:* *: ****. *::**

SP|P02458|CO2A1_HUMAN  CDTGTVLCDDIICEDVKDCLSPEIPFGECCPICPTDLAT-ASGQPGPKGQKGEPGDIKDI 115
SP|P28481|CO2A1_MOUSE  CDTGNVLCDDIICED-PDCLNPEIPFGECCPICPADLAT-ASGKLGPKGQKGEPGDIRDI 114
SP|Q91717|CO2A1_XENLA  CDTGTVLCDDIICEESKDCPNAEIPFGECCPICPTEQSSTSSGQGVLKGQKGEPGDIKDV 120
TR|Q2LDA1|Q2LDA1_DANRE CDSGTVLCDEVICEDLNDCANPIISPGECCPICPADTDD-PIGSLGAKGQKGEPGDITDV 119
                       **:*.****::***:  ** .  *  ********::      *.   ********** *:

SP|P02458|CO2A1_HUMAN  VGPKGPPGPQGPAGEQGPRGDRGDKGEKGAPGPRGRDGEPGTPGNPGPPGPPGPPGPPGL 175
SP|P28481|CO2A1_MOUSE  IGPRGPPGPQGPAGEQGPRGDRGDKGEKGAPGPRGRDGEPGTPGNPGPAGPPGPPGPPGL 174
SP|Q91717|CO2A1_XENLA  LGPRGPPGPQGPSGEQGSRGERGDKGEKGAPGPRGRDGEPGTPGNPGPV---GPPGPPGL 177
TR|Q2LDA1|Q2LDA1_DANRE VGPRGPAGPMGPPGEQGTRGERGAKGEKGSPGPRGRDGEPGTPGNPGPPGPPGPNGPPGL 179
                       :**:** ** ** **** **:** *****:******************    ** *****

SP|P02458|CO2A1_HUMAN  G-GNFAAQMAGGFDEKAGGAQLGVMQGPMGPMGPRGPPGPAGAPGPQGFQGNPGEPGEPG 234
SP|P28481|CO2A1_MOUSE  SAGNFAAQMAGGYDEKAGGAQMGVMQGPMGPMGPRGPPGPAGAPGPQGFQGNPGEPGEPG 234
SP|Q91717|CO2A1_XENLA  G-GNFAAQMTGGFDEKAGGAQMGVMQGPMGPMGPRGPPGPTGAPGPQGFQGNPGEPGEPG 236
TR|Q2LDA1|Q2LDA1_DANRE G-GNFAAQMAGGFDEKAGGAQMGVMQGPMGPMGPRGPPGPSGAPGPQGFQGNPGETGEPG 238
                       . *******:**:********:******************:************** ****

SP|P02458|CO2A1_HUMAN  VSGPMGPRGPPGPPGKPGDDGEAGKPGKAGERGPPGPQGARGFPGTPGLPGVKGHRGYPG 294
SP|P28481|CO2A1_MOUSE  VSGPMGPRGPPGPAGKPGDDGEAGKPGKSGERGLPGPQGARGFPGTPGLPGVKGHRGYPG 294
SP|Q91717|CO2A1_XENLA  AGGPMGPRGPPGPSGKPGDDGEAGKPGKSGERGPPGPQGARGFPGTPGLPGVKGHRGYPG 296
TR|Q2LDA1|Q2LDA1_DANRE PAGALGPRGPPGPPGKPGSDGEAGKPGKAGERGPPGPQGARGFPGTPGLPGIKGHRGHPG 298
```

```
                                 .* :******** ****.*********:**** ****************:*****:**

SP|P02458|CO2A1_HUMAN  LDGAKGEAGAPGVKGESGSPGENGSPGPMGPRGLPGERGRTGPAGAAGARGNDGQPGPAG 354
SP|P28481|CO2A1_MOUSE  LDGAKGEAGAPGVKGESGSPGENGSPGPMGPRGLPGERGRTGPAGAAGARGNDGQPGPAG 354
SP|Q91717|CO2A1_XENLA  LDGAKGEAGAAGAKGEGGATGEAGSPGPMGPRGLPGERGRPGSSGAAGARGNDGLPGPAG 356
TR|Q2LDA1|Q2LDA1_DANRE LDGAKGEAGAAGAKGEAGSNGESGAPGPMGPRGLPGERGRPGATGAAGARGNDGLPGPAG 358
                       ********** *.***.*: ** *:*************** * :********** *****

SP|P02458|CO2A1_HUMAN  PPGPVGPAGGPGFPGAPGAKGEAGPTGARGPEGAQGPRGEPGTPGSPGPAGASGNPGTDG 414
SP|P28481|CO2A1_MOUSE  PPGPVGPAGGPGFPGAPGAKGEAGPTGARGPEGAQGSRGEPGNPGSPGPAGASGNPGTDG 414
SP|Q91717|CO2A1_XENLA  PPGPVGPAGAPGFPGAPGSKGEAGPTGARGPEGAQGPRGESGTPGSPGPAGASGNPGTDG 416
TR|Q2LDA1|Q2LDA1_DANRE PPGPVGPAGAPGFPGSPGSKGEAGPTGARGPEGAQGPRGEAGTPGSPGPAGASGNPGTDG 418
                       *********.*****:**:**************** *** *.*****************

SP|P02458|CO2A1_HUMAN  IPGAKGSAGAPGIAGAPGFPGPRGPPGPQGATGPLGPKGQTGEPGIAGFKGEQGPKGEPG 474
SP|P28481|CO2A1_MOUSE  IPGAKGSAGAPGIAGAPGFPGPRGPPGPQGATGPLGPKGQAGEPGIAGFKGDQGPKGETG 474
SP|Q91717|CO2A1_XENLA  IPGAKGSSGGPGIAGAPGFPGPRGPPGPQGATGPLGPKGQTGDPGVAGFKGEQGPKGEIG 476
TR|Q2LDA1|Q2LDA1_DANRE IPGAKGSAGASGIAGAPGFPGPRGPPGPQGATGPLGPKGQSGDPGIPGFKGEAGPKGERG 478
                       *******:*. ****************************:*:**: ****: ***** *

SP|P02458|CO2A1_HUMAN  PAGPQGAPGPAGEEGKRGARGEPGGVGPIGPPGERGAPGNRGFPGQDGLAGPKGAPGERG 534
SP|P28481|CO2A1_MOUSE  PAGPQGAPGPAGEEGKRGARGEPGGAGPIGPPGERGAPGNRGFPGQDGLAGPKGAPGERG 534
SP|Q91717|CO2A1_XENLA  SAGPQGAPGPAGEEGKRGARGEPGAAGPNGPPGERGAPGNRGFPGQDGLAGPKGAPGERG 536
TR|Q2LDA1|Q2LDA1_DANRE VLGPQGPPGPSGEEGKRGPRGEPGSAGPLGPPGERGAPGNRGFPGQDGLAGAKGAPGDRG 538
                         **** ***:******* *****..** ********************* *****:**

SP|P02458|CO2A1_HUMAN  PSGLAGPKGANGDPGRPGEPGLPGARGLTGRPGDAGPQGKVGPSGAPGEDGRPGPPGPQG 594
SP|P28481|CO2A1_MOUSE  PSGLTGPKGANGDPGRPGEPGLPGARGLTGRPGDAGPQGKVGPSGAPGEDGRPGPPGPQG 594
SP|Q91717|CO2A1_XENLA  VPGLGGPKGGNGDPGRPGEPGLPGARGLTGRPGDAGPQGKVGPSGASGEDGRPGPPGPQG 596
TR|Q2LDA1|Q2LDA1_DANRE VPGLSGPKGGTGDPGRPGEPGLPGARGLTGRPGDAGAQGKVGATGAPGEDGRPGPPGPLG 598
                         ** ****..************************ ***** :** *********** *

SP|P02458|CO2A1_HUMAN  ARGQPGVMGFPGPKGANGEPGKAGEKGLPGAPGLRGLPGKDGETGAAGPPGPAGPAGERG 654
SP|P28481|CO2A1_MOUSE  ARGQPGVMGFPGPKGANGEPGKAGEKGLAGAPGLRGLPGKDGETGAAGPPGPSGPAGERG 654
SP|Q91717|CO2A1_XENLA  ARGQPGVMGFPGPKGANGEPGKAGEKGLVGAPGLRGLPGKDGETGSQGPNGPAGPAGERG 656
TR|Q2LDA1|Q2LDA1_DANRE ARGQPGVMGFPGPKGANGEPGKPGEKGLVGRTGLRGLPGKDGETGPSGPPGPVGAVGERG 658
                       ********************* ***** *  ************  ** ** * .****

SP|P02458|CO2A1_HUMAN  EQGAPGPSGFQGLPGPPGPPGEGGKPGDQGVPGEAGAPGLVGPRGERGFPGERGSPGAQG 714
SP|P28481|CO2A1_MOUSE  EQGAPGPSGFQGLPGPPGPPGEGGKQGDQGIPGEAGAPGLVGPRGERGFPGERGSPGAQG 714
SP|Q91717|CO2A1_XENLA  EQGPPGPSGFQGLPGPPGSPGEGGKPGDQGVPGEAGAPGLVGPRGERGFPGERGSSGPQG 716
```

```
TR|Q2LDA1|Q2LDA1_DANRE EQGQPGPSGFQGLPGPTGAPGEPGKPGDQGVPGEGGAAGPTGPRGERGFPGERGGAGPQG 718
                       *** ************ * *** ** ****:***.** *  .*************. * **

SP|P02458|CO2A1_HUMAN  LQGPRGLPGTPGTDGPKGASGPAGPPGAQGPPGLQGMPGERGAAGIAGPKGDRGDVGEKG 774
SP|P28481|CO2A1_MOUSE  LQGPRGLPGTPGTDGPKGAAGPDGPPGAQGPPGLQGMPGERGAAGIAGPKGDRGDVGEKG 774
SP|Q91717|CO2A1_XENLA  LQGPRGLPGTPGTDGPKGASGPSGPNGAQGPPGLQGMPGERGAAGISGPKGDRGDTGEKG 776
TR|Q2LDA1|Q2LDA1_DANRE LQGPRGLPGTPGTDGPKGAIGPAGAAGAQGPPGLQGMPGERGAVGISGAKGDRGDSGEKG 778
                       ******************* ** *  *****************.**:* ****** ****

SP|P02458|CO2A1_HUMAN  PEGAPGKDGGRGLTGPIGPPGPAGANGEKGEVGPPGPAGSAGARGAPGERGETGPPGPAG 834
SP|P28481|CO2A1_MOUSE  PEGAPGKDGGRGLTGPIGPPGPAGANGEKGEVGPPGPSGSTGARGAPGERGETGPPGPAG 834
SP|Q91717|CO2A1_XENLA  PEGASGKDGSRGLTGPIGPPGPAGPNGEKGESGPSGPPGIVGARGAPGDRGENGPPGPAG 836
TR|Q2LDA1|Q2LDA1_DANRE PEGAPGKDGSRGLTGPIGPPGPSGPNGAKGETGPIGSIGAPGARGAPGDRGEIGAPGPAG 838
                       **** ****.************:* ** *** ** *  *  *******:*** * *****

SP|P02458|CO2A1_HUMAN  FAGPPGADGQPGAKGEQGEAGQKGDAGAPGPQGPSGAPGPQGPTGVTGPKGARGAQGPPG 894
SP|P28481|CO2A1_MOUSE  FAGPPGADGQPGAKGDQGEAGQKGDAGAPGPQGPSGAPGPQGPTGVTGPKGARGAQGPPG 894
SP|Q91717|CO2A1_XENLA  FAGPPGADGQSGLKGDQGESGQKGDAGAPGPQGPSGAPGPQGPTGVFGPKGARGAQGPAG 896
TR|Q2LDA1|Q2LDA1_DANRE FAGPPGADGQPGNKGEQGESGQKGDSGAPGPQGPSGAPGPVGPTGVTGPKGARGAQGAPG 898
                       ********** * **:***:*****:************** ***** **********  *

SP|P02458|CO2A1_HUMAN  ATGFPGAAGRVGPPGSNGNPGPPGPPGPSGKDGPKGARGDSGPPGRAGEPGLQGPAGPPG 954
SP|P28481|CO2A1_MOUSE  ATGFPGAAGRVGPPGANGNPGPAGPPGPAGKDGPKGVRGDSGPPGRAGDPGLQGPAGAPG 954
SP|Q91717|CO2A1_XENLA  ATGFPGAAGRVGTPGPNGNPGPPGPPGSAGKEGPKGVRGDAGPPGRAGDPGLQGAAGAPG 956
TR|Q2LDA1|Q2LDA1_DANRE ATGFPGAAGRVGPPGPNGNPGAAGPAGPSGKDGPKGVRGDAGPPGRAGDAGLRGPPGAPG 958
                       ************ ** *****  ** * :**:****.***:*******: **:*  * **

SP|P02458|CO2A1_HUMAN  EKGEPGDDGPSGAEGPPGPQGLAGQRGIVGLPGQRGERGFPGLPGPSGEPGKQGAPGASG 1014
SP|P28481|CO2A1_MOUSE  EKGEPGDDGPSGLDGPPGPQGLAGQRGIVGLPGQRGERGFPGLPGPSGEPGKQGAPGASG 1014
SP|Q91717|CO2A1_XENLA  EKGEPGEDGPSGPDGPPGPQGLSGQRGIVGLPGQRGERGFPGLPGPSGEPGKQGGPGSSG 1016
TR|Q2LDA1|Q2LDA1_DANRE EKGEAGEDGPPGPDGPSGPAGLAGQRGIVGLPGQRGERGFPGLPGPSGEPGKQGAPGGSG 1018
                       **** *:*** * :** ** **:*******************************.**.**

SP|P02458|CO2A1_HUMAN  DRGPPGPVGPPGLTGPAGEPGREGSPGADGPPGRDGAAGVKGDRGETGAVGAPGAPGPPG 1074
SP|P28481|CO2A1_MOUSE  DRGPPGPVGPPGLTGPAGEPGREGSPGADGPPGRDGAAGVKGDRGETGALGAPGAPGPPG 1074
SP|Q91717|CO2A1_XENLA  DRGPPGPVGPPGLTGPSGEPGREGNPGSDGPPGRDGATGIKGDRGETGPLGAPGAPGAPG 1076
TR|Q2LDA1|Q2LDA1_DANRE DRGPPGPVGPPGLTGPAGETGREGNPGSDGPPGRDGAAGVKGERGNTGPIGAPGAPGAPG 1078
                       ****************:** ****.**:*********:*:**:**:** :******* **

SP|P02458|CO2A1_HUMAN  SPGPAGPTGKQGDRGEAGAQGPMGPSGPAGARGIQGPQGPRGDKGEAGEPGERGLKGHRG 1134
SP|P28481|CO2A1_MOUSE  SPGPAGPTGKQGDRGEAGAQGPMGPSGPAGARGIAGPQGPRGDKGESGEQGERGLKGHRG 1134
```

```
SP|Q91717|CO2A1_XENLA  APGSVGPTGKQGDRGESGPQGPLGPSGPAGARGLAGPQGPRGDKGEAGEAGERGQKGHRG 1136
TR|Q2LDA1|Q2LDA1_DANRE APGSVGPIGKQGDRGENGPQGPAGPPGPAGARGMVGPQGPRGDKGEAGEAGERGQKGHRG 1138
                       :** .** ******** * *** ** *******: ***********:** **** *****

SP|P02458|CO2A1_HUMAN  FTGLQGLPGPPGPSGDQGASGPAGPSGPRGPPGPVGPSGKDGANGIPGPIGPPGPRGRSG 1194
SP|P28481|CO2A1_MOUSE  FTGLQGLPGPPGPSGDQGASGPAGPSGPRGPPGPVGPSGKDGSNGIPGPIGPPGPRGRSG 1194
SP|Q91717|CO2A1_XENLA  FTGLQGLPGPPGSAGDQGATGPAGPAGPRGPPGPVGPSGKDGSNGISGPIGPPGPRGRSG 1196
TR|Q2LDA1|Q2LDA1_DANRE FTGLQGLPGPPGSPGDQGAAGPAGPSGAKGPSGPVGPAGKDGSNGQPGPIGPPGPRGRSG 1198
                       ************  *****:*****:* :** *****:****:**  *************

SP|P02458|CO2A1_HUMAN  ETGPAGPPGNPGPPGPPGPPGPGIDMSAFAGLGPREKGPDPLQYMRADQAAGGLRQHDAE 1254
SP|P28481|CO2A1_MOUSE  ETGPVGPPGSPGPPGPPGPPGPGIDMSAFAGLGQREKGPDPMQYMRADEADSTLRQHDVE 1254
SP|Q91717|CO2A1_XENLA  ETGPSGPPGQPGPPGPPGPPGPGIDMSAFAGLSQPEKGPDPMRYMRADQASNSL---PVD 1253
TR|Q2LDA1|Q2LDA1_DANRE ESGPVGPPGNPGPPGPPGPPGPGIDMSAFAGLSQPEKGPDPLRYMRADEASSSLRQHDVE 1258
                       *:** ****.**********************.  ******::*****:* . *    .:

SP|P02458|CO2A1_HUMAN  VDATLKSLNNQIESIRSPEGSRKNPARTCRDLKLCHPEWKSGDYWIDPNQGCTLDAMKVF 1314
SP|P28481|CO2A1_MOUSE  VDATLKSLNNQIESIRSPDGSRKNPARTCQDLKLCHPEWKSGDYWIDPNQGCTLDAMKVF 1314
SP|Q91717|CO2A1_XENLA  VEATLKSLNNQIENIRSPDGTKKNPARTCRDLKLCHPEWKSGDYWIDPNQGCTVDAIKVF 1313
TR|Q2LDA1|Q2LDA1_DANRE VDATLKSINGQIEDIRSPDGSRKNPARSCRDLKLCHPEWKSGDYWVDPNLGSAADAIKVF 1318
                       *:*****:*.***.****:*::*****:*:***************:*** *.: **:***

SP|P02458|CO2A1_HUMAN  CNMETGETCVYPNPANVPKKNWWSSKSKEKKHIWFGETINGGFHFSYGDDNLAPNTANVQ 1374
SP|P28481|CO2A1_MOUSE  CNMETGETCVYPNPATVPRKNWWSSKSKEKKHIWFGETMNGGFHFSYGDGNLAPNTANVQ 1374
SP|Q91717|CO2A1_XENLA  CDMETGETCVYPNPSKIPKKNWWSAKGKEKKHIWFGETINGGFQFSYGDDSSAPNTANIQ 1373
TR|Q2LDA1|Q2LDA1_DANRE CNMETGETCVKPSTPKIPRKNWWTSKSKAQKHVWFGESMNGGFHFSYADGSQTPSTTTIQ 1378
                       *:******** *.  .:*:****::*.* :**:****::****:***.*.. :*.*:.:*

SP|P02458|CO2A1_HUMAN  MTFLRLLSTEGSQNITYHCKNSIAYLDEAAGNLKKALLIQGSNDVEIRAEGNSRFTYTAL 1434
SP|P28481|CO2A1_MOUSE  MTFLRLLSTEGSQNITYHCKNSIAYLDEAAGNLKKALLIQGSNDVEMRAEGNSRFTYTAL 1434
SP|Q91717|CO2A1_XENLA  MTFLRLLSTDASQNITYHCKNSIAFMDEASGNLKKAVLLQGSNDVEIRAEGNSRFTYNAL 1433
TR|Q2LDA1|Q2LDA1_DANRE LNFLRLLSTEATQTITYHCKNSVAYMDQATGNLKKAILLQGSNDVEIRAEGNSRFTYGVL 1438
                       :.*******:.:*.********:*::*:*:******:*:*******:********** .*

SP|P02458|CO2A1_HUMAN  KDGCTKHTGKWGKTVIEYRSQKTSRLPIIDIAPMDIGGPEQEFGVDIGPVCFL 1487
SP|P28481|CO2A1_MOUSE  KDGCTKHTGKWGKTVIEYRSQKTSRLPIIDIAPMDIGGAEQEFGVDIGPVCFL 1487
SP|Q91717|CO2A1_XENLA  EDGCKKHTGKWSKTVIEYRTQKTSRLPIVDIAPMDIGGADQEFGVDIGPVCFL 1486
TR|Q2LDA1|Q2LDA1_DANRE EDGCKKHTGQWAKTVIEYKTQKTSRLPIMDIAPMDIGGADQEFGVDIGAVCFL 1491
                       :***.****:*.******::********:********* :******** ****
```

Protein sequence alignment of COL2A1 protein. COL2A1 proteins show high conservation of the primary structure among distant species. The figure shows CLUSTAL OMEGA (1.2.4) multiple sequence alignment of the regions of COL2A1 protein of different organisms: Human, Mus musculus, Xenopus laevis, and Danio rerio [Uniprot identifiers: P02458, P28481, Q91717, Q2LDA, respectively]. Highlighted amino acids show the mutations found in Perthes patients. c.638 G > A (p.Gly213Asp) (exon 9); c.2014 G > T (p.Gly672Cys) (exon 31) (NM_001844.5) [50]; c.1888 G > A (p.Gly630Ser) (exon 29) (NM_033150.3) [51]; c.3665 G > A (p.Gly1170Ser) (exon 50) (NM_033150.3) [48,49].

48. Su, P.; Li, R.; Liu, S.; Zhou, Y.; Wang, X.; Patil, N.; Mow, C.S.; Mason, J.C.; Huang, D.; Wang, Y. Age at onset-dependent presentations of premature hip osteoarthritis, avascular necrosis of the femoral head, or Legg-Calvé-Perthes disease in a single family, consequent upon a p.Gly1170Ser mutation of COL2A1. *Arthritis Rheum.* **2008**, *58*, 1701–1706.
49. Miyamoto, Y.; Matsuda, T.; Kitoh, H.; Haga, N.; Ohashi, H.; Nishimura, G.; Ikegawa, S. A recurrent mutation in type II collagen gene causes Legg-Calvé-Perthes disease in a Japanese family. *Hum. Genet.* **2007**, *121*, 625–629. https://doi.org/10.1007/s00439-007-0354-y.
50. Kannu, P.; Irving, M.; Aftimos, S.; Savarirayan, R. Two Novel COL2A1 Mutations Associated with a Legg-Calvé-Perthes Disease-like Presentation. *Clin. Orthop. Relat. Res.* **2011**, *469*, 1785–1790. https://doi.org/10.1007/s11999-011-1850-x.
51. Li, N.; Yu, J.; Cao, X.; Wu, Q.-Y.; Li, W.-W.; Li, T.-F.; Zhang, C.; Cui, Y.-X.; Li, X.-J.; Yin, Z.-M.; et al. A novel p. Gly630Ser mutation of COL2A1 in a Chinese family with presentations of Legg-Calvé-Perthes disease or avascular necrosis of the femoral head. *PLoS ONE* **2014**, *9*, e100505.
